# The Influence Mechanism of Ettringite Crystals and Microstructure Characteristics on the Strength of Calcium-Based Stabilized Soil

**DOI:** 10.3390/ma14061359

**Published:** 2021-03-11

**Authors:** Youmin Han, Junwu Xia, Hongfei Chang, Jun Xu

**Affiliations:** 1 State Key Laboratory for Geomechanics and Deep Underground Engineering, China University of Mining and Technology, Daxue Road, Xuzhou 221116, China; hanyoumin@ahpu.edu.cn (Y.H.); honfee@126.com (H.C.); 2 School of Architecture and Civil Engineering, Anhui Polytechnic University, Beijing Road, Wuhu 241000, China; 3 Jiangsu Collaborative Innovation Center for Building Energy Saving and Construction Technology, Xueyuan Road, Xuzhou 221116, China; 4 School of Civil Engineering and Architecture, Jiangsu University of Science and Technology, Changhui Road, Zhenjiang 212100, China; xujun@just.edu.cn; 5 School of Materials Science and Engineering, Southeast University, Southeast University Road, Nanjing 211189, China

**Keywords:** calcium-based stabilized soil, unconfined compressive strength, hydration product, ettringite, calcium silicate hydrate, microstructure characteristic

## Abstract

To reveal the influence mechanism of ettringite (AFt) crystals and microstructure characteristics on the strength of calcium-based stabilized soil, the strengths and microscopic properties of seven groups of stabilized soil samples were studied systematically through unconfined compressive strength, scanning electron microscope (SEM), X-ray diffraction (XRD), thermogravimetry (TG), and Fourier transform infrared spectroscopy (FTIR) testing methods. The results indicate that the strength of the cement-stabilized soil is relatively high because abundant calcium silicate hydrate (CSH) gels coat the outer surface of soil particles to cement together. For the cement–gypsum-stabilized soil, superabundant thick and long AFt crystals make the pores in soil particles larger, and the sample becomes looser, resulting in lower strength than that of the cement-stabilized soil. However, the strength of the cement–gypsum–lime-stabilized soil is slightly stronger than that of the cement-stabilized soil, for the reason that the appropriate amount of fine AFt crystals fill the macropores between soil particles to form a network space structure and sufficient CSH gels cement the soil particles and the AFt crystals network space structure tightly together. It could be suggested that the components of calcium-based stabilizer should consider the optimal production balance between CSH gels and fine AFt crystals.

## 1. Introduction

With the implementation of the development strategy for coastal, riverside, and lakeside areas in various countries, many large-scale infrastructure construction projects need to be launched in these areas. Soft soil will inevitably be encountered in these areas. Soft soil stabilization technology is the most traditional method for soft soil treatment in the construction of buildings, tunnels, roads, railways, airports, etc. Therefore, the study of soft soil stabilizers has become one of the hot topics of civil engineering research [1].

The purpose of soft soil stabilization is to improve the properties such as compressive strength, deformation, moisture absorption, and California bearing ratio, among which the compressive strength is the key characteristic parameter of stabilized soil.

The most conventional soft soil stabilizers in engineering are calcium-based stabilizers, which are rich in calcium minerals such as ordinary Portland cement, lime, and part of industrial wastes. The stabilization mechanism of the cement-stabilized soil mainly comes from the hydrolysis and hydration reaction of cement, followed by the ion-exchange reaction and agglomeration, pozzolanic reaction, and carbonation reaction between soil particles and cement hydrate [2]. When lime [2] or carbide slag [3,4] is used as a soft soil stabilizer, its stabilization mechanism originates from the ion-exchange reaction, pozzolanic reaction, and carbonation reaction between calcium hydroxide and soil particles. Industrial slag [5,6], fly ash [7,8], natural pozzolans [9], zeolite [10], municipal solid waste incineration fly ash [11], and other pozzolanic materials can also be applied to a stabilizer. The activated silica and alumina contained in these pozzolanic materials undergo a pozzolanic reaction in the alkaline environment of calcium hydroxide. According to the microstructural analysis such as scanning electron microscope (SEM) and X-ray diffraction (XRD), the main hydration products of calcium-based stabilized soil include calcium silicate hydrate (CSH) gels, calcium aluminate hydrate (CAH) crystals, calcium hydroxide (CH) crystals, calcium carbonate (CaCO_3_), etc. The strength of calcium-based stabilized soil mainly comes from CSH gels and CaCO_3_.

In some studies, gypsum [8], recycled bassanite [12], or phosphogypsum [7] was added into cement and lime to form a new compound stabilizer, which significantly increases the formation amount of ettringite (AFt) crystals in hydration products. AFt crystals are recognized as unfavorable products in concrete due to their high expansiveness, which induces concrete cracking when they are formed [13]. However, there are two completely different conclusions about the role of AFt crystals in stabilized soil. One is that the adverse effects caused by the formation of AFt crystals should be avoided [5], and the other is that the generation of AFt crystals can fill the pores in soil particles to improve the strength of stabilized soil [12,14]. Given the above, the influence mechanism of AFt crystals on the strength of calcium-based stabilized soil is still unclear. The cooperative working mechanism of AFt crystals and CSH gels will be discussed further in this article.

In this paper, seven groups of calcium-based stabilized soil samples were formed by single-doped stabilizer, double-doped stabilizer, and three-doped stabilizer with the raw materials of cement, gypsum, and lime. The strengths of the stabilized soil samples were obtained through the unconfined compressive strength (UCS) test. In the meantime, the microscopic properties of the stabilized soil samples were studied systematically by analysis means of SEM, XRD, thermogravimetry (TG), and Fourier transform infrared spectroscopy (FTIR). By comparative analysis of the strengths, hydration products, and microstructure characteristics of the stabilized soil samples, the influence mechanism of AFt crystals and microstructure characteristics on the strength of calcium-based stabilized soil was revealed. The main influencing factors for the strength of calcium-based stabilized soil and the selection basis of each component of calcium-based stabilizer were summarized, which can provide a certain theoretical basis for the application of calcium-based stabilizer in soft soil stabilization.

## 2. Materials and Methods

### 2.1. Soils and Stabilizers

The test soil in this paper was collected from the third clay layer of the foundation ditch of the Innovation Training Center for College Students of China University of Mining and Technology. The original clay was air-dried, crushed, sifting through a 2-mm sieve, and finally sealed and bagged as the test soil. The characteristic indexes of the tested soil, such as the pH value, specific gravity, plastic limit, liquid limit, and particle size distribution, were measured and shown in Table 1.

The raw materials of the stabilizers include ordinary Portland cement (PC) with cube compressive strength not less than 42.5 MPa at 28 days, gypsum, and lime. X-ray fluorescence (XRF) analysis was performed to obtain the mass percentages of oxide composition of soil, cement, gypsum, and lime sifted through a 200-mesh sieve, as shown in Table 2.

### 2.2. Sample Preparation

There are seven groups of stabilized soil samples formed by three groups of single-doped stabilizer, three groups of double-doped stabilizer, and one group of three-doped stabilizer with the raw materials of cement, gypsum, and lime in the test. The seven groups are listed as follows: the cement-stabilized soil sample (PC100S), the gypsum-stabilized soil sample (G100S), the lime-stabilized soil sample (L100S), the cement–gypsum-stabilized soil sample (PC70G30S), the cement–lime-stabilized soil sample (PC70L30S), the gypsum–lime-stabilized soil sample (G80L20S) and the cement–gypsum–lime-stabilized soil sample (PC70G24L6). The raw material components and their mass percentages of the stabilized soil samples are shown in Table 3. Since the engineering background of the research was the soil reinforcement of building foundation and underground engineering, stabilizer contents, water contents of soil samples, and curing ages of all stabilized soil samples were 16%, 80%, and 28 days, respectively.

According to the test design in Table 3, the amount of soil, water, and all components in the stabilizer for each group of samples were calculated and prepared. The soil and all components in the stabilizer were manually mixed in the stirring pot, and then water was added and stirred evenly with a mixer. After the stabilized soil slurry had a certain viscosity, it was poured into 50 mm × 50 mm × 50 mm cubic test molds coated with a vaseline release agent and vibrated on a small vibrating table to shape samples. There were three samples in each group. Each sample was labeled with a corresponding number and covered with plastic film. It should be noted that the indoor environment and water temperature were 23 °C, and the relative humidity was 72%. After 24 h, all samples were demoulded, wrapped in plastic film, and placed in a standard curing chamber with an ambient temperature of 20 ± 2 °C and relative humidity of 95 ± 5% until the designed age. Since G100S failed to form, it was directly put into the standard curing chamber without demoulding.

### 2.3. Testing Methods

The testing methods of the stabilized soil samples are shown in Table 3.

#### 2.3.1. Unconfined Compressive Strength (UCS)

The UCS test was carried out by a 20-kN electronic universal testing machine (Docer CSS-88020, Jinan, China). The test loading speed was 1 mm/min.

One of each group of crushed samples was soaked in anhydrous ethanol for seven days to terminate its hydration reaction, which was applied to subsequent SEM, XRD, TG, and FTIR tests [15,16].

#### 2.3.2. Scanning Electron Microscope (SEM)

SEM analysis is a type of microstructure imaging technology that takes advantage of the properties of the sample surface materials. The SEM (FEI Quanta^TM^ 250, Austin, TX, USA) was adopted for scanning electron microscopy. The samples were dried in the oven at a temperature of 60 °C and then cut into 7 mm × 7 mm × 7 mm cubes with a blade. The test surface must be a relatively flat fresh surface that was exposed by manual opening along the nicks. The back of the fresh surface adhered to the bracket with double-sided adhesive tape, and then the fresh surface was sprayed with gold to improve the conductivity of the sample surface [17]. After the above test preparation process is completed, the SEM image acquisition in high vacuum mode can be carried out. The magnifications of the images collected in this paper are all 8000 times.

#### 2.3.3. X-Ray Diffraction (XRD)

XRD analysis is a kind of technology that utilizes the diffraction effect of X-ray in crystal materials to analyze the structure of materials. It can be applied to the qualitative and semi-quantitative analysis of crystal phases. The X-ray diffractometer (Bruker D8 ADVANCE, Karlsruhe, Germany) was used for phase diffraction analysis. The samples were dried for 6 h in the oven at 60 °C and crushed into small pieces. Then they were ground into powders with agate mortar and sifted through a 200-mesh sieve [17]. Each group of samples was bagged with no less than 1 g of powder for diffraction analysis.

#### 2.3.4. Thermogravimetry–Derivative Thermogravimetry (TG–DTG)

TG analysis is a technique for measuring the relationship between weight and temperature under a program-controlled temperature. The derivative thermogravimetric (DTG) curve represents the relationship between the change rate of weight and temperature, which is the first derivative of the TG curve to temperature and also the peak curve of the thermal weight loss rate. The synchronous thermal analyzer (Mettler Toledo TGA/DSC1/1100LF, Zurich, Switzerland) was used for thermogravimetric analysis. The samples are prepared by the same process as in sub-Section 2.3.3. Each group of samples was bagged with no less than 0.5 g powder for thermogravimetric analysis.

#### 2.3.5. Fourier Transform Infrared Spectroscopy (FTIR)

FTIR analysis is a kind of technique that makes use of the resonance of each group in the molecule with the infrared spectrum of the same vibration frequency and then forms the infrared absorption spectrum that represents the structural characteristics of the material molecule. It is usually applied to analyze the composition of the molecule and the types of chemical bonds. The Fourier transform infrared spectrometer (Bruker VERTEX 80v, Karlsruhe, Germany) was adopted for infrared spectrum analysis. The sample preparation process was the same as that in sub-Section 2.3.3. Each group of samples was packed with no less than 1 g powder for infrared spectroscopy analysis.

## 3. Experimental Results

### 3.1. UCS Results

**Figure 1:**
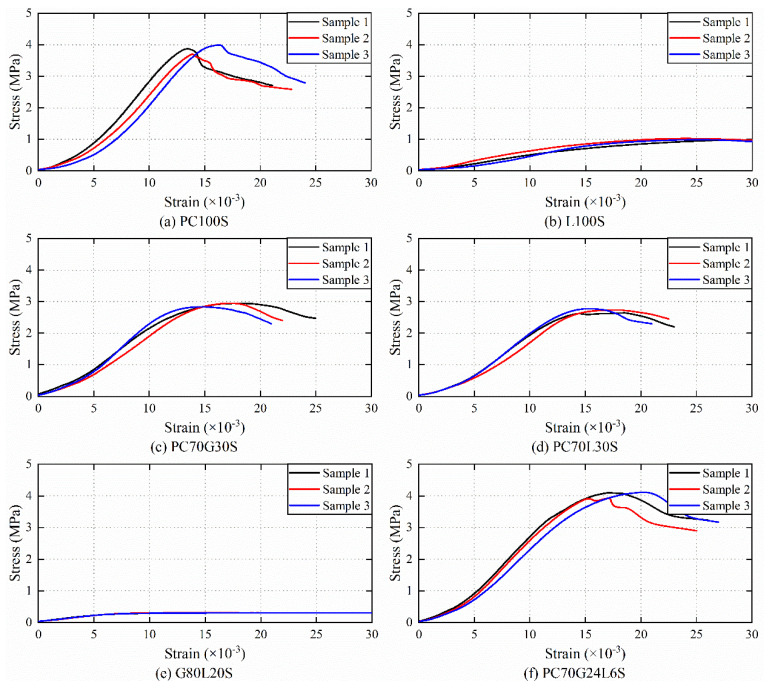
Stress–strain curves of the stabilized soil samples.

Since G100S failed to form after 28 days of curing, there was no compressive strength data. The stress–strain curves of the other six groups of stabilized soil samples are shown in Figure 1.

**Figure 2:**
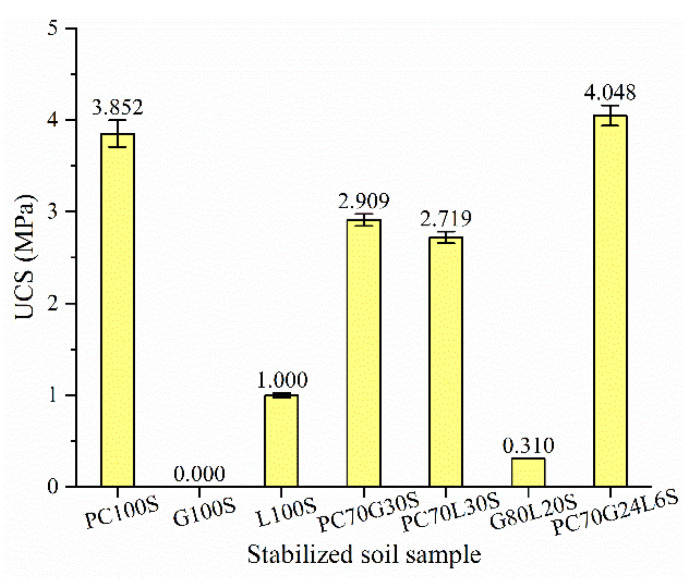
Unconfined compressive strength (UCS) of the stabilized soil samples.

The UCS average value of three samples of each group of stabilized soil was taken as the compressive strength of the group of samples, which is shown in Figure 2. The compressive strength of L100S is about 25.96% of that of PC100S. It indicates that the cement-stabilized soil has the highest compressive strength, followed by the lime-stabilized soil among three groups of single-doped stabilized soils. The gypsum-stabilized soil cannot be formed.

The compressive strength of PC70G30S, PC70L30S, and G80L20S are 75.52%, 70.59%, and 8.05% of that of PC100S, respectively, that is to say, the compressive strength of three groups of double-doped stabilized soil is lower than that of the cement-stabilized soil. It shows that the double-doped stabilizers have no more contribution to improve the compressive strength of stabilized soils than cement.

The compressive strength of PC70G24L6 is 105.09% of that of PC100S, i.e., the compressive strength of the three-doped stabilized soil mixed with cement, gypsum, and lime at the same time is higher than that of any single-doped or double-doped stabilized soil. It illustrates that the compressive strength of stabilized soil can be improved as an appropriate amount of gypsum and lime are added into cement to form a three-doped stabilizer.

### 3.2. SEM Analysis

**Figure 3:**
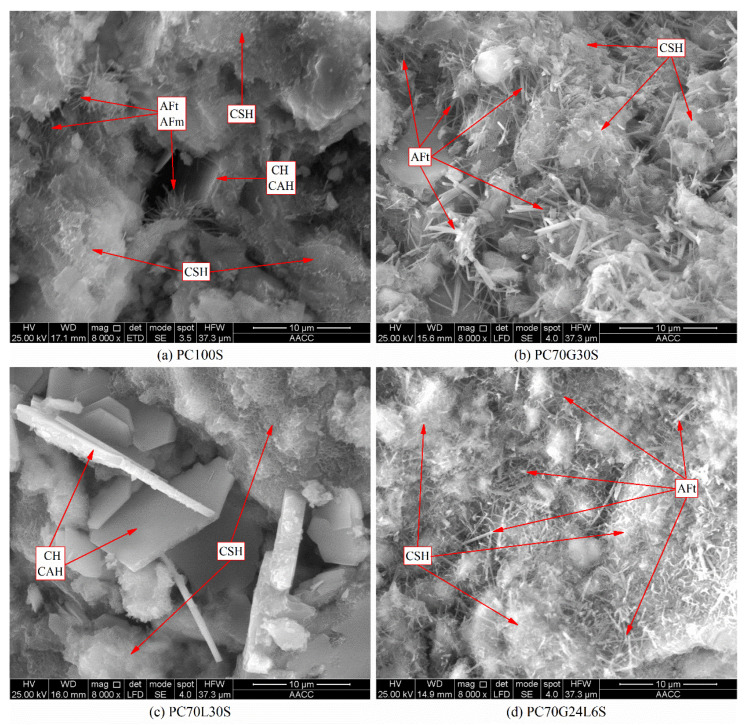
Scanning electron microscope (SEM) images of the stabilized soil samples.

The microstructure properties of PC100S, PC70G30S, PC70L30S, and PC70G24L6 were analyzed by SEM images. The phase morphology and structural composition characteristics of the stabilized soil samples are shown in Figure 3.

As shown in Figure 3a, abundant flocculated CSH gels [10,11,18,19,20,21,22] were generated in PC100S, which coated the outer surface of soil particles and cemented the soil particles together to form a whole structure. However, some large pores can still be observed. Moreover, a little of needle-like AFt crystals and monosulfate calcium sulfoaluminate hydrate (AFm) crystals [10,11,12,18,20,21,23] can be detected in the pores, together with individual hexagonal plate-shaped CH crystals [18,19,20,21] and cubic-shaped CAH crystals [20]. The AFt crystals were about 1–2 μm in length and 0.1 μm in diameter. The number of AFt crystals was too few to fill large pores.

From Figure 3b, it can be known that many flocculated CSH gels and needle-shaped AFt crystals were generated in PC70G30S [12], but no obvious hexagonal plate-shaped CH crystals and cubic-shaped CAH crystals were found. The AFt crystals were about 2~5 μm in length and 0.1–0.5 μm in diameter. The size of AFt crystals in PC70G30S was much larger than that in PC100S. For the large difference in scale between CSH gels and AFt crystals, they were almost independent of each other. Superabundant thick and long AFt crystals made the pores in soil particles larger, while CSH gels cannot cement the soil particles and the AFt crystals together to form a whole structure. Therefore, this sample became relatively loose.

It can be observed from Figure 3c that many flocculated CSH gels and hexagonal plate-shaped CH crystals and cubic-shaped CAH crystals were generated in PC70L30S, but no obvious needle-shaped AFt crystals were found. The CH crystals and CAH crystals were about 3~10 μm in length and 0.3~1 μm in thickness. They were mixed between the soil particles wrapped in CSH gels, resulting in large pores.

As shown in Figure 3d, many flocculated CSH gels and needle-shaped AFt crystals were generated also in PC70G24L6, and no obvious hexagonal plate-shaped CH crystals and cubic-shaped CAH crystals were observed. The AFt crystals were about 1~3 μm in length and 0.1~0.2 μm in diameter. The size of AFt crystals was slightly larger than that in PC100S but much smaller than that in PC70G30S. The AFt crystals filled the large pores between soil particles and interweaved with each other, forming a network space structure and making the pore size relatively smaller. Moreover, the CSH gels wrapped the outer surface of the soil particles and the AFt crystals network space structure. The cementation of CSH gels made the soil particles and the AFt crystals network space structure form a relatively compact whole structure.

### 3.3. XRD Analysis

**Figure 4:**
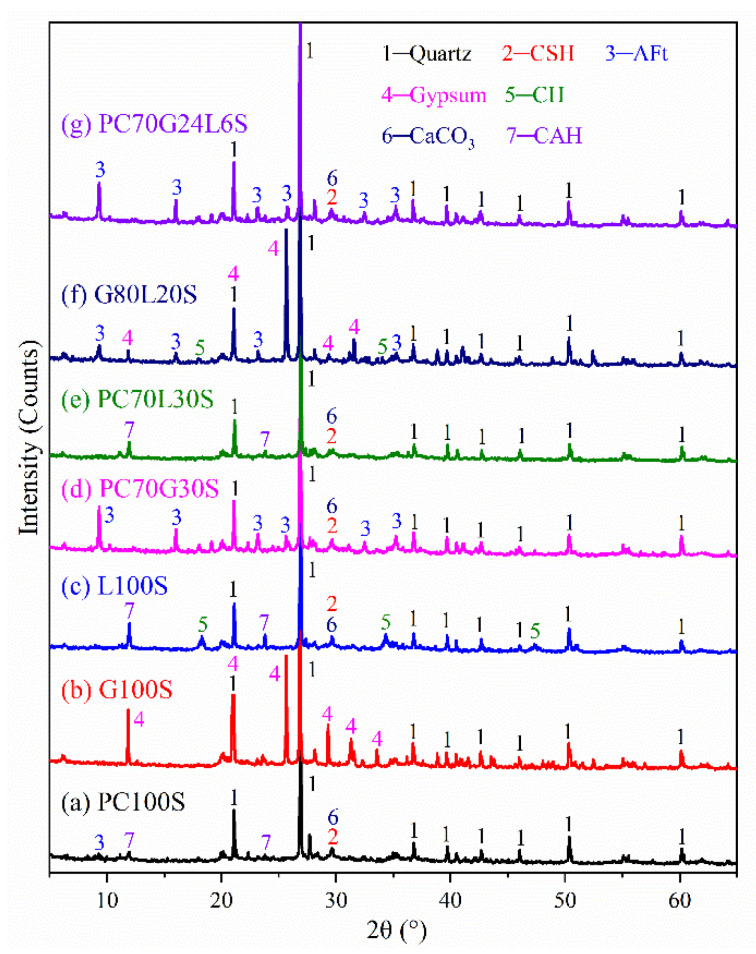
X-ray diffraction (XRD) diffraction patterns of the stabilized soil samples.

XRD analysis was conducted to obtain the composition of the seven stabilized soil samples, as shown in Figure 4. There were very strong quartz peaks in the seven samples [12]. Quartz is the main component in the original soil, which will not be described later.

As shown in Figure 4a, there were weak CSH gel peaks, CaCO_3_ peaks, AFt crystal peaks, and CAH crystal peaks for PC100S [2,11]. As a result of the poor crystallinity of CSH gel, small XRD peaks of CSH gel do not mean a small amount of CSH gels. The content determination of CSH gels should be analyzed synthetically through SEM and TG–DTG.

In addition, there were very strong gypsum peaks for G100S (Figure 4b) [24]. Similarly, there were very strong gypsum peaks, together with weak AFt crystal peaks and CH crystal peaks for G80L20S (Figure 4f) [12].

As for L100S (Figure 4c) and PC70L30S (Figure 4e), there were strong CAH crystal peaks, combined with weak CaCO_3_ peaks and CSH gel peaks. L100S also had strong CH crystal peaks [2,3].

Furthermore, for PC70G30S (Figure 4d) [12] and PC70G24L6S (Figure 4g), there were very strong AFt crystal peaks, and weak CSH gel peaks and CaCO_3_ peaks. According to the XRD results alone, there is little difference between PC70G30S and PC70G24L6S. Therefore, a comprehensive analysis combining with XRD and SEM is in need.

### 3.4. TG–DTG Analysis

**Figure 5:**
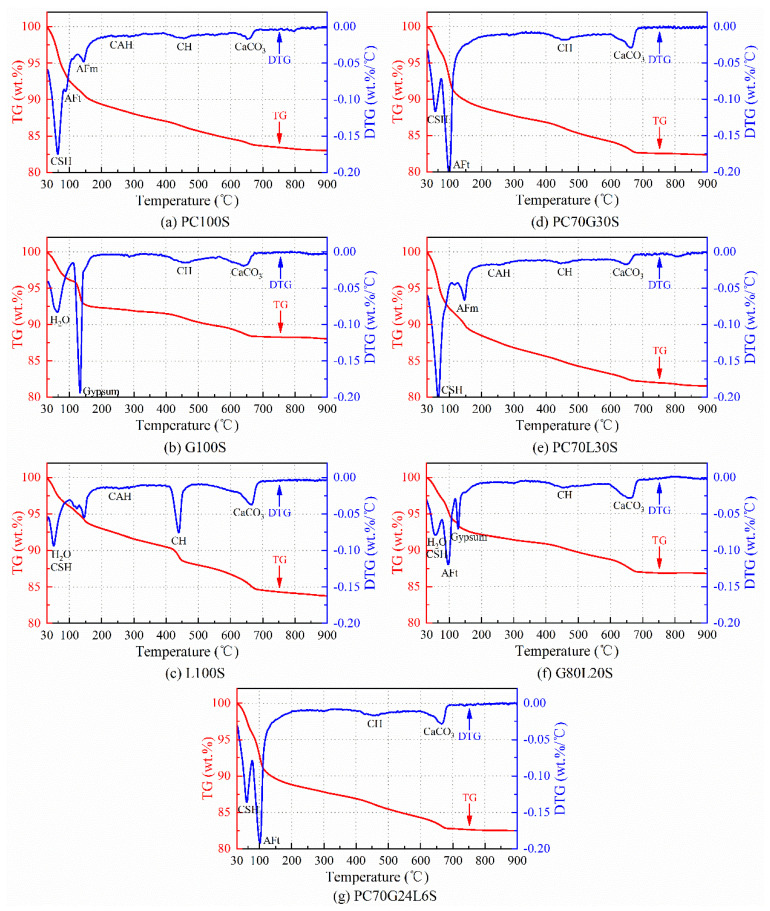
Thermogravimetry and derivative thermogravimetry (TG–DTG) curves of the stabilized soil samples.

The TG and DTG curves of the seven stabilized soil samples are shown in Figure 5. The peaks in DTG curves correspond to the mass-loss rates during the thermal decompositions.

For the PC100S sample (Figure 5a), a strong peak was observed at the temperature of 65 °C, which was detected as the mass loss of pore adsorption water in CSH gels [15,25]. Two weak peaks corresponding to crystal water for AFt crystals and AFm crystals appeared at around 90 °C and 145 °C, respectively [24,26]. Another two weak peaks emerged at around 450 °C and 650 °C, which corresponded to dehydration of CH crystals and decarbonation of CaCO_3_, respectively [15,24,25,27]. In addition, at about 260 °C, a very weak peak may exist, which was related to the escape of bound water for CAH crystals [3,24]. From Figure 5a,e, it is found that there is little difference between PC70L30S and PC100S. However, PC70L30S had a stronger peak of pore adsorption water in CSH gels without the peak of crystal water for AFt crystals being observed.

As can be derived from Figure 5b, G100S had a weak peak of pore adsorption water at about 63 °C [24] and a very sharp peak of crystal water in dihydrate gypsum crystals at about 133 °C [26]. For the L100S sample (Figure 5c), there was a weak peak of pore adsorption water at about 52 °C [24], which might contain a small amount of pore adsorption water in CSH gels. At around 440 °C and 660 °C, there was a sharp dehydration peak of CH crystals and a decarbonation peak of CaCO_3_, respectively. There might be a very weak peak of bound water for CAH crystals at about 260 °C [3]. For the G80L20S sample (Figure 5f), there was a weak peak of pore adsorption water at about 58 °C, which might contain a little pore absorbed water in CSH gels. There was a slightly strong peak of crystal water for AFt crystals at about 97 °C and a weak peak of crystal water for dihydrate gypsum crystals at 128 °C, without the peak of crystal water for AFm crystals being found.

Furthermore, for PC70G30S (Figure 5d) and PC70G24L6 (Figure 5g), a slightly strong peak of pore adsorption water in CSH gels occurred at around 57 °C, and a very sharp peak of crystal water for AFt crystals occurred at 97 °C without the peak of crystal water for AFm crystals being observed. From the results of TG–DTG, there is little difference between PC70G30S and PC70G24L6. However, the peak value of pore adsorption water in CSH gels of PC70G24L6 was slightly larger than that of PC70G30S and smaller than that of PC100S, whereas the peak value of crystal water for AFt crystals of PC70G24L6 was slightly smaller than that of PC70G30S and obviously larger than that of PC100S.

### 3.5. FTIR Analysis

**Figure 6:**
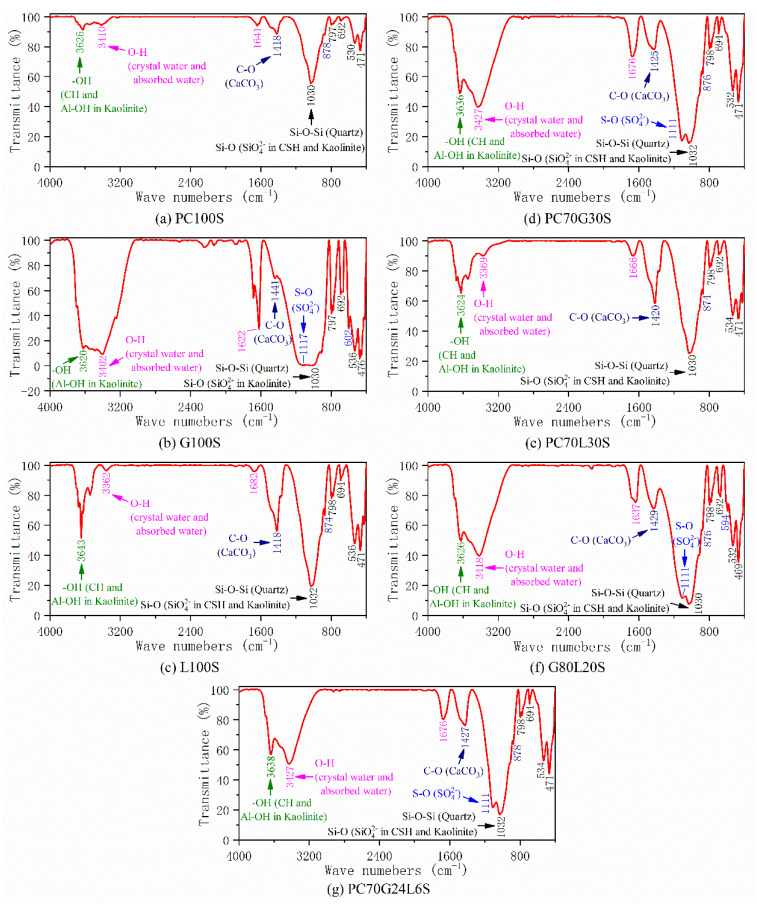
Fourier transform infrared spectroscopy (FTIR) spectrograms of the stabilized soil samples.

The functional groups in the components of the seven stabilized soil samples were obtained by FTIR test, and the results are shown in Figure 6. Since the main component of the seven samples was soil, the test results of each sample contained the adsorption band of Si–O–Si tetrahedron in quartz. The Si–O absorption band of SiO_4_^2−^ tetrahedral anion and the –OH absorption band of Al–OH octahedron in kaolin were also contained. The adsorption bands are listed as follows: the overlapped antisymmetric stretching vibration absorption bands (1030 cm^−1^) of Si–O–Si in quartz and Si–O in kaolin, the divided symmetric stretching vibration absorption bands (798 cm^−1^ and 780 cm^−1^) of Si–O–Si in quartz, the asymmetric flexural vibration absorption band (694 cm^−1^) of Si–O–Si in quartz, the symmetric flexural vibration absorption band (471 cm^−1^) of Si–O–Si in quartz, the asymmetric flexural vibration absorption band (534cm^−1^) of Si–O in kaolin, and the stretching vibration absorption band (3626 cm^−1^) of –OH in kaolin [2,28,29].

As can be observed from Figure 6a, as a result of the influence of the absorption bands of quartz and kaolin in the PC100S sample, the Si–O absorption band of SiO_4_^2−^ ion in CSH gel and the –OH absorption band in CH crystals could not be separated from them. The wavenumbers of 1030 cm^−1^ and 530 cm^−1^ might partly belong to the Si–O absorption band of SiO_4_^2−^ ion in CSH gels, and the wavenumber of 3626 cm^−1^ might partly belong to the –OH adsorption band in CH crystals [2,9,13,29]. There was a weak antisymmetric stretching vibration adsorption band (1418 cm^−1^) and an out-of-plane flexural vibration adsorption band (878 cm^−1^) of planar tetratomic CO_3_^2−^ ion in CaCO_3_ [2,9,13,29], in addition to a weak stretching vibration absorption band (3410 cm^−1^) and a flexural vibration absorption band (1641 cm^−1^) of O–H in crystal water and pore adsorption water for CSH gels, AFt crystals and AFm crystals [13,24,28].

As for G100S (Figure 6b), on account of the existence of a large amount of gypsum, there was a very strong antisymmetric stretching vibration absorption band (1117 cm^−1^) and an asymmetric flexural vibration absorption band (602 cm^−1^) of S–O in SO_4_^2−^ ion [13,24], together with very strong O–H absorption bands (3402 cm^−1^ and 1622 cm^−1^) of crystal water and pore absorbed water in gypsum crystals [24,28].

For L100S (Figure 6c), the presence of a large amount of lime led to very sharp absorption bands of –OH (3643cm^−1^) in CH crystals and CO_3_^2−^ ion (1418 cm^−1^ and 874 cm^−1^) in CaCO_3_ [13]. Similarly, Figure 6e shows that PC70L30S also had stronger absorption bands of –OH (3624 cm^−1^) in CH and CO_3_^2−^ ion (1420 cm^−1^ and 874 cm^−1^) in CaCO_3_ than PC100S due to the addition of lime to cement.

As for PC70G30S (Figure 6d), G80L20S (Figure 6f), and PC70G24L6S (Figure 6g), there are strong absorption bands of S–O (1111 cm^−1^ and 594 cm^−1^) in SO_4_^2−^ ion, O–H (3427 cm^−1^ and 1676 cm^−1^) in crystal water and pore absorbed water, –OH (3636 cm^−1^) in CH, and CO_3_^2−^ ion (1425 cm^−1^ and 876 cm^−1^) in CaCO_3_. From the results of FTIR alone, there is little difference between PC70G30S, G80L20S, and PC70G24L6; therefore, it is necessary to combine SEM, TG–DTG, and XRD for analysis.

## 4. Discussion

According to the above test results, the microscopic test methods of SEM, XRD, TG–DTG, and FTIR have different sensitivity to the characterization of various phases. A single test method cannot make a valid judgment, whereas the results of various test methods complement and verify each other to further determine the hydration products and microstructure characteristics of various stabilized soils. Through the comparative analysis of strengths, hydration products, and microstructure characteristics of the stabilized soils, the influence mechanism of hydration products and microstructure characteristics on the strength of calcium-based soil was discussed as follows.

The hydration products of the cement-stabilized soil (PC100S) are mainly CSH gels (Figure 5a). The SEM image (Figure 3a) shows that a large number of CSH gels coat the outer surface of the soil particles, cementing the soil particles together. A few AFt crystals, AFm crystals, CH crystals, CAH crystals, and CaCO_3_ are contained in some larger pores (Figure 5a). The strength of the cement-stabilized soil mainly depends on CSH gels, AFt crystals, and CaCO_3_, while AFm crystals, CH crystals, and CAH crystals have little contribution to the strength. The hydration products and microstructure characteristics of the cement-stabilized soil determine its high strength.

There is almost no hydration reaction between gypsum and soil in the gypsum-stabilized soil (G100S). The final main components are still mainly soil and gypsum, especially dihydrate gypsum and anhydrous gypsum Figure 4b and Figure 5b). Therefore, after 28 days, it still did not form and had no compressive strength. Due to the microporous properties of gypsum, a large amount of absorbed water is contained in the micropores, which has been verified by TG–DTG and FTIR analysis (Figure 5b and Figure 6b).

A large number of CH crystals were found in the lime-stabilized soil (L100S) (Figure 5c and Figure 6c), accompanied by a small amount of CSH gels, CAH crystals, and CaCO_3_ (Figure 4c, Figure 5c, and Figure 6c). The compressive strength of the lime-stabilized soil is much lower than that of the cement-stabilized soil (Figure 2). This is because CH in the solution reacts with partially active SiO_2_ and Al_2_O_3_ in soil particles to produce CSH and CAH after lime hydrolysis. CH can also absorb CO_2_ in water and air to produce water-insoluble CaCO_3_. A small amount of CSH gels and CaCO_3_ are the main contributors to the strength of the lime-stabilized soil, while CH crystals and CAH crystals have little contribution to strength.

The hydration products of the cement–gypsum-stabilized soil (PC70G30S) are still mainly CSH gels (Figure 3b and Figure 5d), together with numerous thick and long AFt crystals (Figure 3b, Figure 4d, Figure 5d and Figure 6d). AFt crystals are formed by the reaction of gypsum with tricalcium aluminate (C_3_A) and tetracalcium aluminoferrite (C_4_AF) in cement. Superabundant thick and long AFt crystals make the pores in soil particles larger, while CSH gels cannot cement the soil particles and the AFt crystals together, and hence the stabilized soil becomes looser (Figure 3b). As a result, its strength is slightly lower than that of the cement-stabilized soil (Figure 2).

It can be derived that the hydration products of the cement–lime-stabilized soil (PC70L30S) are still dominated by CSH gels (Figure 3c and Figure 5e), together with many CH crystals (Figure 3c and Figure 6e), CAH crystals (Figure 4e), and CaCO_3_ (Figure 6e). The addition of lime promotes the hydration reaction rate of cement (Figure 5a,e). However, CH crystals and CAH crystals reduce the strength of stabilized soil, leading to a slightly lower strength than that of the cement-stabilized soil (Figure 2).

There is a large amount of gypsum found in the gypsum–lime-stabilized soil (G80L20S) (Figure 4f and Figure 5f). Moreover, there are a few CSH gels and AFt crystals generated by pozzolan reaction of lime, gypsum together with active SiO_2_ and Al_2_O_3_ in the soil particles (Figure 4f and Figure 5f), and also a few CaCO_3_ generated by carbonation reaction of lime (Figure 6f). Since only a small amount of CSH gels, AFt crystals, and CaCO_3_ can provide a little bit of strength, the strength of the gypsum–lime-stabilized soil is very low (Figure 2).

As can be derived from the SEM images in Figure 3b,d, the hydration products of the cement–gypsum–lime-stabilized soil (PC70G24L6S) are almost the same as those of the cement–gypsum-stabilized soil. The results of XRD, TG–DTG, and FTIR also show the same conclusion (Figure 4d,g, Figure 5d,g and Figure 6d,g). However, the hydration reaction of cement and the generation of AFt are promoted because of the addition of lime. The production of CSH gels is slightly higher (Figure 5d,g), and the size of AFt crystals is relatively smaller (Figure 3b,d) [30]. The appropriate amount of fine AFt crystals filled the large pores between soil particles and formed a network space structure. However, they do not increase the pore size between the soil particles as the larger AFt crystals do in the cement–gypsum-stabilized soil. Therefore, the macropores amount in the cement–gypsum–lime-stabilized soil decreases. Moreover, sufficient CSH gels wrap the outer surface of the soil particles and the AFt crystals network space structure. The cementation of CSH gels makes the soil particles and the AFt crystals network space structure form a relatively compact whole structure (Figure 3d); therefore, the strength of the cement–gypsum–lime-stabilized soil is significantly stronger than that of the cement–gypsum-stabilized soil (Figure 2).

Furthermore, comparing the cement–gypsum–lime-stabilized soil and the cement-stabilized soil, although the production of CSH gels in the former reduces (Figure 5a,g), its macropores amount decreases dramatically for the macropores are filled by the fine AFt crystals (Figure 3a,d); thus, the former has a slightly stronger strength than the latter (Figure 2). It should be noted that the microstructure characteristic of AFt crystals is one of the main influencing factors for the strength of the calcium-based stabilized soil.

In summary, the two main influencing factors for the strength of the calcium-based stabilized soil are the production of CSH gels and the content of harmful pores (macropores). The content of harmful pores mainly depends on the production and size of AFt crystals. Superabundant thick and long AFt crystals make the pores in soil particles bigger and reduce the strength of stabilized soil. On the contrary, the appropriate amount of fine AFt crystals can fill the macropores between soil particles and form a network space structure, which is beneficial to improve the strength of stabilized soil. As a result, the proportion of each component in calcium-based stabilizer should consider the production of CSH gels and fine AFt crystals in the stabilized soil to achieve an optimal balance.

## 5. Conclusions

The strengths and microscopic properties of seven calcium-based stabilized soils were systematically studied through UCS, SEM, XRD, TG, and FTIR testing methods. The main conclusions are as follows:

(1) In three groups of single-doped stabilized soils, the compressive strength of the cement-stabilized soil is the highest, followed by the lime-stabilized soil, and the gypsum-stabilized soil cannot be shaped. For three groups of double-doped stabilized soils, namely, the cement–gypsum-stabilized soil, the cement–lime-stabilized soil, and the gypsum–lime-stabilized soil, their compressive strength is lower than that of the cement-stabilized soil, indicating that the double-doped stabilizers have no more contribution to improve the compressive strength of stabilized soil than cement. However, the compressive strength of the cement–gypsum–lime-stabilized soil is higher than that of any single-doped or double-doped stabilized soils, showing that the compressive strength of stabilized soil can be improved as an appropriate amount of gypsum and lime are added into cement at the same time to form a three-doped stabilizer;
(2) For the cement-stabilized soil, abundant CSH gels coat the outer surface of soil particles to make them cemented together. A few AFt crystals filled in macropores have little effect on improving the strength. Therefore, the strength of the cement-stabilized soil is relatively high but still lower than the cement–gypsum–lime-stabilized soil;
(3) As for the cement–gypsum–stabilized soil, superabundant thick and long AFt crystals make the pores in soil particles larger, while CSH gels cannot cement the soil particles and the AFt crystals together, and hence the stabilized soil becomes looser. As a result, its strength is slightly lower than that of the cement-stabilized soil;
(4) For the cement–gypsum–lime-stabilized soil, the appropriate amount of fine AFt crystals fills the macropores between soil particles and form a network space structure. Sufficient CSH gels wrap the outer surface of the soil particles and the AFt crystals network space structure to make them cemented tightly together. Therefore, its strength is slightly stronger than that of the cement-stabilized soil and far stronger than that of the cement–gypsum–stabilized soil;
(5) The two main influencing factors for the strength of the calcium-based stabilized soil are the production of CSH gels and the content of harmful pores (macropores). The content of harmful pores mainly depends on the production and size of AFt crystals. Superabundant thick and long AFt crystals make the pores in soil particles larger, which decreases the strength of stabilized soil, whereas the appropriate amount of fine AFt crystals filling the macropores between soil particles and forming a network space structure improve the strength of stabilized soil. Therefore, the proportion of each component in calcium-based stabilizer should consider the production of CSH gels and fine AFt crystals to achieve an optimal balance.

The research provides a theoretical basis for the optimization of stabilizers for soft soil with constant water content. Of course, the influence of AFt crystals and microstructure characteristics on deformation, moisture absorption, and California bearing ratio of stabilized soil needs further study, and the application range can be extended from soft soil with constant water content to any other type of soft soil.

## Figures and Tables

**Table 1:** Characteristic indexes of the tested soil.

Index ^1^	Value
pH	7.92
Specific gravity, *G_s_*	2.75
Plastic limit, *W_P_* (%)	24.1
Liquid limit, *W_L_* (%)	44.2
Grain size distribution (%)	—
Clay (<0.002 mm)	9.15
Silt (0.002–0.075 mm)	80.28
Sand (0.075–2 mm)	10.57

^1^ Measured as per GB/T 50123-2019 (China MOHURD).

**Table 2:** Oxide composition of the tested soil and each component of the stabilizers.

Oxide Composition ^1^	Content by Mass (%)			
	Soil	PC	Gypsum	Lime
SiO_2_	64.47	19.83	1.38	0.28
Al_2_O_3_	15.86	7.75	0.08	0.13
CaO	1.22	52.42	35.85	73.83
SO_3_	0.02	3.70	45.59	0.24
Fe_2_O_3_	5.72	3.97	0.03	0.28
MgO	1.32	2.25	3.38	0.50
K_2_O	2.14	0.77	—	0.01
TiO_2_	0.80	0.34	—	—
Na_2_O	0.55	0.18	—	—
Others	0.03	0.31	0.39	0.01
Loss on ignition ^2^	7.87	8.48	13.30	24.72

^1^ Oxide composition was analyzed by an X-ray fluorescence (XRF) spectrometer (Bruker S8 TIGER, Karlsruhe, Germany). ^2^ The loss on ignition measured at 950 °C.

**Table 3:** Test grouping details of stabilized soil samples.

Stabilized Soil Sample	The Proportion of Each Component in Stabilizer (%)			UCS	SEM	XRD	TG–DTG	FTIR
	PC	Gypsum	Lime					
PC100S	100	—	—	√	√	√	√	√
G100S	—	100	—	√		√	√	√
L100S	—	—	100	√		√	√	√
PC70G30S	70	30	—	√	√	√	√	√
PC70L30S	70	—	30	√	√	√	√	√
G80L20S	—	80	20	√		√	√	√
PC70G24L6S	70	24	6	√	√	√	√	√

## Data Availability

All data reported in this paper is contained within the manuscript.
